# A Maximum Dose Bioassay to Assess Efficacy of Spinetoram against Cowpea Thrip *Megalurothrips usitatus* in China

**DOI:** 10.3390/insects15060412

**Published:** 2024-06-03

**Authors:** Huailiang Yu, Mingyue Wu, Shaoka Li, Jin Li, Xiang Zou, Zhaojiang Guo, Qingjun Wu, Youjun Zhang, Xiangyi Kong, Wen Xie

**Affiliations:** 1 Tropical Specialty Fruit and Vegetable Research Lab, Sanya Academy of Tropical Agricultural Sciences, Sanya 572022, China; aleyn1196@foxmail.com (H.Y.); wumingyuecaas2021@163.com (M.W.); 18789327911@163.com (S.L.); z179012191@163.com (X.Z.); 2 School of Tropical Agriculture and Forestry, Hainan University, Haikou 570228, China; 3 Institute of Tropical Fruit Trees, Hainan Academy of Agricultural Sciences, Haikou 571100, China; 4 Sanya Institute, China Agricultural University, Sanya 572025, China; lijin2017@163.com; 5 Department of Plant Protection, Institute of Vegetables and Flowers, Chinese Academy of Agricultural Sciences, Beijing 100081, China; guozhaojiang@caas.cn (Z.G.); wuqingjun@caas.cn (Q.W.); zhangyoujun@caas.cn (Y.Z.); 6 National Nanfan Research Institute (Sanya), Chinese Academy of Agricultural Sciences, Sanya 572024, China

**Keywords:** cowpea thrip, *Megalurothrips usitatus* (Bagnall), spinetoram, maximum dose bioassay

## Abstract

**Simple Summary:**

Spinetoram is a semi-synthetic bioinsecticide. This study evaluated the efficacy of spinetoram, the main insecticide against cowpea thrips, on cowpea in the main production areas of Hainan. The maximum dose bioassay method was used to assess field populations of cowpea thrips collected from 20 villages in Yazhou District. The results showed that mortality rates of cowpea thrip populations ranged from 3.31% to 100%, with 66.98% of the populations exceeding 80% mortality and 33.96% exceeding 90% mortality. Significant differences in mortality rates were observed between populations from different villages. In conclusion, the maximum dose bioassay method provided valuable insights into the field efficacy of spinetoram against cowpea thrips and highlighted the importance of caution when using it in combination with other methods to reduce the potential for resistance.

**Abstract:**

The bean flower thrip *Megalurothrips usitatus* (Bagnall) is a severe pest on cowpeas and causes a 20–30% reduction in cowpeas in Hainan, China, with even complete crop failure in severe cases. Spinetoram is currently the most important pesticide against *M. usitatus* in cowpea production. In the main producing areas of cowpeas in Hainan, however, the efficacy of spinetoram against *M. usitatus* is not well known. In the present study, we employed the maximum dose bioassay to evaluate the efficacy of the mortality rates of adult thrips at F_0_ in spinetoram, freshly collected from 212 field populations of *M. usitatus* collected from 20 villages in the Yazhou District of Hainan. Our results showed that the mortality rates of these thrip populations exposed to spinetoram were from 3.31% to 100%. Among them, the mortality rates of 66.98% (142/212) of the populations exceeded 80%, while that of 33.96% (72/212) of the populations surpassed 90%. Only a small proportion of 0.47% (1/212) the populations exhibited a mortality rate below 10%, and 4.72% (10/212) displayed rates below 50%. Furthermore, significant differences were also observed in the mortality rates of thrips among different villages. Taken together, the maximum dosage bioassay method is a rapid and easily implemented approach providing valuable insights into the field efficacy of insecticides and offers guidance in determining the optimal dosage required in the field. Spinetoram is still effective against *M. usitatus* in the main producing areas of cowpeas in Hainan, but caution should be exercised in its combined use with other methods to reduce potential resistance.

## 1. Introduction

Cowpea (*Vigna unguiculata* L. Walp.), belonging to the genus Vigna [1,2], is a species of annual herbaceous plant in the legume family [3]. Cowpea is the main variety cultivated in most parts of China [4]. According to the statistical yearbook of 2022, in Hainan Province in China, the sown area of cowpeas reached 23,077 hectares, with a total production of 582,100 metric tons, of which 80% was sold inland [5]. During the winter season, Sanya, located in the southernmost part of Hainan, has indeed emerged as a significant “vegetable basket” supplier for the entire country [6]. The planting area of cowpeas in Yazhou District accounts for more than half of the total in Sanya [7] because this area’s favorable climate and fertile soil conditions have made it an ideal location for the cultivation of cowpea. 

Although the hot and humid environment in Hainan provides favorable conditions for yardlong beans, it also contribute to the proliferation of diverse pests infesting yardlong beans [8]. Among the handful of pest species, thrips are particularly the dominant pest, resulting in a 20–30% reduction in cowpea yield, and in severe cases, the reduction can reach up to 80% or even complete crop failure [9,10,11,12]. Four thrip species, *Megalurothrips usitatus* (Bagnall), *Frankliniella intonsa* (Trybom), *Thrips palmi* (Karny), and *Thrips hawaiiensis* (Morgan), were found on cowpeas [13], among which *M. usitatus* is the dominant species in most cowpea-producing areas in China [14]. 

Spinetoram, a semi-synthetic bioinsecticide developed by Corteva Agriscience (Corteva Agriscience LLC, Indianapolis, IN, USA) in the 1990s, possesses broad-spectrum, high-efficiency, low-toxicity, and low-residue characteristics [15]. It is strongly evidenced that spinetoram shows high efficacy against thrip pests [9,16,17,18,19,20]. However, in China, thrips in different provinces have shown mild resistance to spinetoram. It is also important to avoid the misuse of methods that result in a rapid increase in resistance. For example, since 2017, certain regions in Hainan have begun to demonstrate moderate resistance to spinetoram [9,21,22,23]. In other regions of China, spinetoram remains effective against thrips, albeit with a rising trend in resistance. During the peak season of sugarcane thrips in Guangxi, the application of 60 g/L spinetoram effectively controlled the infestation [24]. Similarly, in Shaanxi, the same concentration of spinetoram proved effective against grape thrips [25]. However, in Qinghai, *F. occidentalis* specimens collected from chili peppers exhibited reduced sensitivity to spinetoram [26]. Studies conducted in Beijing, Shandong, and Yunnan have revealed varying degrees of resistance to spinetoram among *F. occidentalis* populations [27,28]. Furthermore, *Thrips palmi* and *F. occidentalis* from vegetables like aubergines, chili peppers, and cucumbers in Inner Mongolia, Liaoning, Beijing, Shandong, Zhejiang, Fujian, Guangdong, and Yunnan have developed varying levels of resistance to spinetoram [29]. This suggests that while spinetoram remains a viable option in some areas, its efficacy is being challenged by increasing resistance in multiple regions across China.

The maximum dose bioassay is a swift and straightforward method for biological assessment. It involves utilizing the highest recommended dosage to mimic the actual insecticide application in field conditions, enabling the prediction of insecticides’ potential effectiveness in the field. This bioassay boasts numerous advantages, including its speed, simplicity of setup, and cost efficiency. It not only offers valuable insights into the insecticides’ field performance but also serves as a guide for determining the optimal dosage required in the field. This information can be leveraged to assess insecticide resistance in field populations and aid in planning seasonal insecticide rotations [30,31,32].

The objective of our current research is to address two crucial questions: firstly, to assess the practicality of the maximum dose bioassay method; and secondly, to determine the sustainability of spinetoram’s application against cowpea thrips in the designated region. For this purpose, we conducted a bioassay involving a comprehensive collection of 212 field populations of thrips from the primary bean-growing regions of Yacheng District, Sanya, spanning from November 2022 to March 2023. Utilizing the maximum dose bioassays with spinetoram against these thrips, we aimed to evaluate the resistance levels exhibited by these 212 field populations. The insights gained from this study will inform our decisions on pesticide selection, ensuring a more informed and effective approach to pest management.

## 2. Materials and Methods

### 2.1. Sample Collection

**Figure 1:**
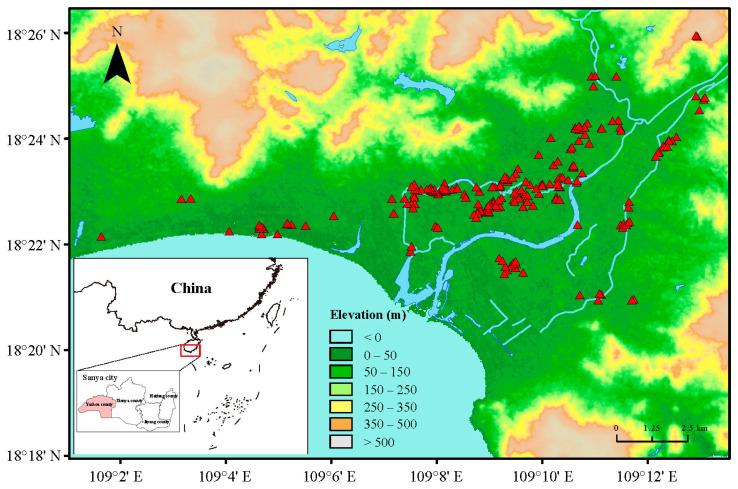
Map of 212 field populations sites (red triangular symbol) collected in Yazhou District, Sanya City, Hainan Province.

From November 2022 to March 2023, a total of 212 field populations of thrips were collected from the flowers of cowpeas in 20 villages of Yazhou District, Sanya City, Hainan Province, China (Table 1 and Table S1, Figure 1). Considering the fact that *M. usitatus* is the dominant species in most cowpea-producing areas in Hainan, we therefore collected *M. usitatus* for further maximum dose bioassays. For each sampling site, a minimum of 50 cowpea flowers containing thrips were randomly collected and placed in an insect-proof net cage as a single sample. 

The laboratory thrip populations were originally collected in 2020 in the experimental field of Sanya Academy of Tropical Agricultural Sciences (18.388681° N, 109.168258° E). Adults were brought back to the laboratory and reared on fresh cowpeas grown without pesticides in self-made glass bottles (flattened drum-shaped bottles with a hole cut in the lid covered by a layer of 200-mesh gauze to prevent thrips from escaping) in an artificial climate chamber (MGC-300H, Yiheng Instruments, Shanghai, China). The rearing conditions were (26 ± 1) °C, relative humidity 70 ± 7%, and L:D = 14:10.

### 2.2. Insecticide

The spinetoram used in this study was a 60 g/L suspension manufactured from Corteva Agriscience (Corteva Agriscience LLC, Indianapolis, IN, USA). The maximum dose was achieved by diluting the cowpea soaking solution according to the highest labeled dosage per ha (750 mL/ha), using a spray volume of 900 L per ha. Based on this, the test dose for the maximum dose bioassay is 0.833 mL/L. The dosage of pesticides used by farmers in the field is defaulted to the recommended label dosage. Distilled water served as the control (Table 2).

### 2.3. Maximum Dose Bioassay

In the present study, we employed the improved leaf membrane tube method to determine the mortality rates of thrip populations exposed to spinetoram. The detailed processes of the bioassays are as follows. The lid of the 3.5 mm diameter petri dish was cracked 2–3 times (for ventilation), a round piece of filter paper (d = 3.5 mm) was placed in the bottom of the dish, and a suitable amount of pesticide was dripped into the petri dish using a rubber-tipped burette to make a film dish and left to dry. Healthy and uncontaminated cowpeas were purchased, washed and dried, cut into 1–1.5 cm pieces (without holes at either end), soaked in the solution for 30 s, removed and allowed to dry naturally at room temperature, then placed in the petri dish. Subsequently, 20–30 freshly collected adult thrips (mixed sexes, tested at F_0_) of each field population were introduced into each dish, while the laboratory-sensitive population served as the control group. The connection between the lid and the dish was sealed with parafilm to prevent thrips from escaping. Each treatment was replicated six times. The treated test thrips were incubated in an artificial climatic chamber for 48 h, and the number of dead and surviving thrips was recorded. Thrips were gently touched with a soft brush tip. They were considered alive if they exhibited normal movement and posture; otherwise, they were considered dead. 

### 2.4. Data Analysis

A total of 35,052 adult thrips were subjected to the bioassay. The data collected from the experiment were recorded in Microsoft Excel 2016 and underwent statistical analysis to calculate both the mortality rate and adjusted mortality rate, where “T” denotes the test group, “C” represents the control group, and “M.R.” represents the mortality rate.

(1) $$Percentage\;of\;mortality=\frac{Number\;of\;dead\;adult}{Number\;of\;adults\;treated}\times 100$$

(2) $$Corrected\;percentage\;of\;mortality=\left(\frac{M.R.of\;T-M.R.of\;C}{1-M.R.of\;C}\right)\times 100$$

The data analysis was performed using the R Programming Language (version 4.3.3, 29 February 2024), and the graphs were visualized using GraphPad Prism (Version 9.5.0, CA, USA). The General Linear Model was used to evaluate the differences between the field populations and the laboratory-susceptible population of thrips across 20 villages and to evaluate the differences between the populations in the village, with a significance level set at *p* < 0.05. The Kruskal–Wallis Test was used to analyze the differences among the sampled points in the same village.

## 3. Results

### 3.1. Inter-Field Populations

Based on the data compilation and analysis, it was found that spinetoram (750 mL/ha, 60 g/L Kodia) remains effective against the cowpea thrips in Sanya, Hainan, China. After evaluating the mortality rates from 212 field populations, it was observed that the highest mortality rate was 100%, while the lowest rate was 3.31% (Table S2). In addition, the mortality rates of 66.98% (142/212) of the populations exceeded 80%, while that of 33.96% (72/212) of the populations surpassed 90%. Only a small proportion of 0.47% (1/212) of the populations exhibited a mortality rate below 10%, and 4.72% (10/212) displayed rates below 50% (Table S2).

### 3.2. Among the Groups from Different Villages

**Figure 2:**
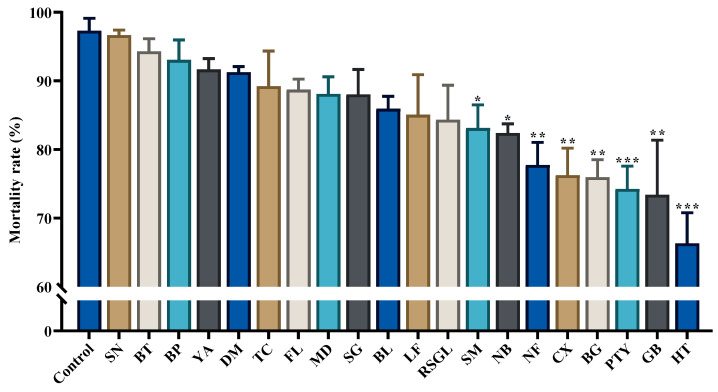
The data in this graph are derived from the mortality rates of thrips in 20 village groups and a susceptible laboratory population. Each data bar represents the mortality rate over a 48 h period exposed to spinetoram or distilled water; ‘***’ indicates that there is a significant difference between treatment and control (*p* < 0.001); ‘**’ indicates that there is a significant difference between treatment and control (*p* < 0.01); ‘*’ indicates the presence of a significant difference between treatment and control (*p* < 0.05); unmarked ‘*’ indicates no significant difference from control.

Based on the analysis results, it was found that spinetoram was effective against thrips. Significant differences were found between the thrip populations from the laboratory and some villages (SM, NB, NF, CX, BG, PTY, GB, and HT; *p* < 0.05; Figure 2; Table 3). When summarizing and sorting the mortality rate of the thrip populations from different villages, it was observed that the thrip populations from 25% (5/20) of the villages had a mortality rate greater than 90%, thrip populations from 70% (14/20) of the villages had a mortality rate greater than 80%, and thrip populations from only 30% (6/20) had a mortality rate lower than 80% (Table S2).

### 3.3. Between the Populations in the Villages

There was no significant difference in the mortality rates between the thrip populations among the BT, DM, NB, YA, or SN villages (Table 3).

## 4. Discussion

The maximum dose bioassay is a valuable tool for assessing the efficacy of insecticides against pests, offering the advantages of being rapid, easy to set up, and cost effective [30,31,32]. We employed the maximum dosage bioassay to evaluate the efficacy of spinetoram against 212 field populations of *M. usitatus* collected from 20 villages in the Yazhou District of Hainan. Our results showed that spinetoram is still effective against *M. usitatus* in the main producing areas of cowpeas in Hainan. Previous results also showed that maximum dose bioassays can be used to assess the efficacy of pesticides against a certain pest in a limited time, which can assist growers in determining whether the pesticide can continue to be used for their comprehensive pest management for the upcoming season. Riley et al. (2020) conducted the maximum dose bioassay to determine several insecticides against the diamondback moth in Georgia and Florida, providing growers with a rapid determination of the insecticidal efficacy against the diamondback moth population on their farms [30]. De Marchi et al. (2021) performed the maximum dose bioassay to assess a variety of insecticides against multiple field populations of whitefly in Florida, thereby enhancing pest management recommendations as part of an integrated management approach. In order to reduce crop damage and increase yields [32], Cremonez et al. (2023) conducted maximum dose bioassays of various insecticides against sweet potato whitefly in pumpkins and cucumbers [31]. In the present study, we employed the maximum dosage bioassay to determine whether spinetoram remains effective against cowpea thrips in Yazhou District, Sanya. Our results showed that the mortality rates of about 67% (142/212) of the thrip populations exceeded 80%, and 33.96% (72/212) of the populations even exceeded 90%, which indicated that spinetoram still remains effective against cowpea thrips in this region. A previous indoor bioassay also indicated a similar result, that spinetoram had high insecticidal activity against cowpea thrips [9,16,19]. On the other hand, apart from cowpea thrips, spinetoram also actually remains effective against thrip pests in other crop systems. For example, Khaliq et al. (2014) demonstrated a noticeable reduction in the population of *Thrips tabaci* on onions following the application of spinetoram [33]. Siebert et al. (2016) found that the application of spinetoram on cotton plants effectively controlled several thrip pests [34].

To address these shortcomings, researchers can integrate the maximum dose bioassay with other techniques, like serial dilution bioassays, to gain a deeper understanding of insecticide effectiveness and resistance monitoring. Regular resistance monitoring is crucial in tracking shifts in pest population sensitivity to insecticides, enabling us to fine-tune insecticide usage strategies accordingly. The maximum dose bioassay can help decision makers in assessing the efficacy of key systemic insecticides used to control pests at multiple sites over a limited period of time. Nevertheless, it is imperative to recognize the limitations of this approach. For instance, the maximum dose bioassay cannot supplant serial dilution bioassays, which yield LC_10_, LC_50_, and LC_90_ values. Furthermore, the elevated doses employed in maximum dose bioassays might overlook resistance. To address these shortcomings, researchers can integrate the maximum dose bioassay with other techniques, like serial dilution bioassays, to gain a deeper understanding of insecticide effectiveness and resistance monitoring. Enhancing experimental design through environmental factor control and employing multiple doses can bolster the method’s precision. Additionally, correlating laboratory outcomes with actual field application impacts is paramount in validating the accuracy of laboratory testing.

Chemical pesticides have become the primary method of pest control, and in some cases, the sole effective control method, due to their high efficiency, rapid effect, and broad spectrum [13]. To mitigate the potential development of thrip resistance to spinetoram, it is imperative to explore alternative, safe, and effective control measures. Zhang et al. (2023b) enhanced the effectiveness of spinetoram against *F*. *intonsa* by incorporating additives [35]. Khan et al. (2023) discovered that specific temperature and humidity conditions synergistically boost spinetoram’s efficacy against *Sitophilus granarius* and *Tribolium castaneum* [36]. Tang et al. (2022) observed a synergistic impact when combining adjuvants with spinetoram in managing cowpea pests [19]. Furthermore, Tang et al. (2016) reported improved efficacy by applying spinetoram prior to the closure of cowpea flowers [37]. Shen et al. (2017) found that combining spinetoram with chlorpyrifos and lambda-cyhalothrin effectively combated common sunflower stem weevils [17]. Wakil et al. (2023) discovered that the combination of *B. bassiana* and spinetoram achieved remarkable results in controlling storage pests [38]. Looking ahead, it is vital for us to meticulously coordinate various measures to combat thrip pests in the Yazhou District of Sanya. Spraying pesticides during the cowpea flowering period is advisable to maximize contact and achieve high efficacy. Adding adjuvants to pesticides and formulating them in conjunction with other pesticides can further enhance their effectiveness and potentially delay the emergence of chemical resistance. Additionally, it is essential to consider the integration of physical, biological, agricultural, and other green control measures in order to construct an IPM technology system and reinforce the pesticide supervision system.

On the other hand, our findings reveal significant differences in mortality rates among the sampled thrip populations within the same village, excluding BT, DM, NB, YA, and SN villages (Table 3). These disparities can be attributed to at least three potential reasons. Firstly, variations in spraying intervals, methods, and the dosages of spinetoram employed may have influenced the results [21]. Secondly, differences in the concentration and frequency of pesticide application likely contributed to the observed differences [22]. Finally, variations in the age and sex ratio of the adult thrips collected for bioassays in different areas within the same and different villages and the implementation of diverse pest control strategies by individual farmers in some villages all may have contributed to the discrepancies in mortality rates among the sampled thrip populations.

## Figures and Tables

**Table 1:** Village group collected in Yazhou District of Sanya in this study.

Population	Village Group	Population Size	Coordinates (E/N)
1	PTY (Potianyang)	39	109.170220 18.391729
2	RSGL (Reshuigong Road)	8	109.166238 18.385317
3	GB (Gongbei)	4	109.163755 18.378950
4	NF (Nanfan)	15	109.160556 18.378576
5	MD (Madan)	19	109.159647 18.381414
6	BT (Batou)	3	109.142166 18.382498
7	SM (Shamai)	18	109.138267 18.384064
8	CX (Chengxi)	9	109.146855 18.376863
9	HT (Haitang)	16	109.135973 18.384014
10	DM (Damao)	7	109.177149 18.403364
11	BL (Beiling)	12	109.182269 18.419855
12	LF (Lifan)	3	109.215252 18.413615
13	BG (Baogu)	9	109.217692 18.412543
14	NB (Nanbin)	13	109.203599 18.395999
15	YA (Yaan)	6	109.195585 18.349518
16	TC (Taice)	4	109.087432 18.373135
17	FL (Fengling)	8	109.083069 18.370051
18	SG (Sangeng)	3	109.052616 18.381189
19	BP (Baoping)	5	109.132956 18.372694
20	SN (Shuinan)	11	109.160618 18.357968

**Table 2:** Background information on the insecticides used in this study.

Insecticide	Formula	Active Ingredient	a.i./ha	Recommended Field Dose ^a^	Test Dose ^b^
Spinetoram	Suspension concentrate (SC)	60 g/L	45 g	600–750 mL/ha	0.833 mL/L

^a^ The recommended field dose according to information from the China Pesticide Information Network: http://www.chinapesticide.org.cn (accessed on 1 December 2023). ^b^ The dose employed in the actual test was derived from the maximum recommended dose.

**Table 3:** The sample size, degrees of freedom, F-statistic, and significance of differences among the points within the groups.

Village Group	N	df	*p*
PTY	39	38	<0.0001
BG	9	8	<0.05
BL	12	11	<0.01
BT	3	2	>0.05
NF	15	14	<0.0001
DM	7	5	>0.05
CX	9	8	<0.0001
GB	4	3	<0.0001
HT	16	15	<0.0001
RSGL	8	7	<0.0001
LF	3	2	<0.05
MD	19	18	<0.0001
SM	18	17	<0.0001
NB	13	12	>0.05
YA	6	5	>0.05
TC	4	3	<0.0001
FL	8	7	<0.05
SG	3	2	<0.05
BP	5	4	<0.0001
SN	11	10	>0.05

The Kruskal–Wallis Test was conducted for each point within each village. Results were considered statistically significant when *p* < 0.05 and highly statistically significant when *p* < 0.01.

## Data Availability

All relevant data are presented in the paper. Additional data can be supplied upon request.

## Supplementary Materials

The following supporting information can be downloaded at https://www.mdpi.com/article/10.3390/insects15060412/s1, Table S1: Field populations collected in Yazhou District of Sanya in this study. Table S2. The mortality rate of 212 field populations and one lab-susceptible population to spinetoram.
