# Supplementary material for: A Maximum Dose Bioassay to Assess Efficacy of Spinetoram against Cowpea Thrip Megalurothrips usitatus in China
Source: Insects. 2024 Jun 3;15(6):412. doi: 10.3390/insects15060412 (PMC11204267; doi:10.3390/insects15060412)
Supplement: Supplementary file 1 [file insects-15-00412-s001.zip › Table S1.pdf]

Table S1. Field populations collected in Yazhou district of Sanya in this study.

Population #	Village group	Population name	Coordinates (E/N)
1	PTY (Potianyang)	PTY1	109.170220 18.391729
2	PTY (Potianyang)	PTY2	109.171506 18.392925
3	PTY (Potianyang)	PTY3	109.175505 18.396743
4	PTY (Potianyang)	PTY4	109.175978 18.397334
5	PTY (Potianyang)	PTY5	109.178236 18.399465
6	PTY (Potianyang)	PTY6	109.180207 18.401251
7	RSGL (Reshuigong Road)	RSGL1	109.166238 18.385317
8	PTY (Potianyang)	PTY7	109.162895 18.385525
9	PTY (Potianyang)	PTY8	109.172326 18.387824
10	PTY (Potianyang)	PTY9	109.181535 18.398503
11	PTY (Potianyang)	PTY10	109.191305 18.403505
12	PTY (Potianyang)	PTY11	109.191544 18.402653
13	RSGL (Reshuigong Road)	RSGL2	109.165701 18.382623
14	GB (Gongbei)	GB1	109.163755 18.378950
15	NF (Nanfan road)	NF1	109.160556 18.378576
16	NF (Nanfan road)	NF2	109.159029 18.390573
17	MD (Madan)	MD1	109.159647 18.381414
18	MD (Madan)	MD2	109.161309 18.382600
19	MD (Madan)	MD3	109.164825 18.384325
20	RSGL (Reshuigong Road)	RSGL3	109.166748 18.385591
21	RSGL (Reshuigong Road)	RSGL4	109.166645 18.385198
22	PTY (Potianyang)	PTY12	109.169048 18.385757
23	PTY (Potianyang)	PTY13	109.171746 18.387377
24	PTY (Potianyang)	PTY14	109.172980 18.387798
25	PTY (Potianyang)	PTYI5	109.174077 18.370861
26	PTY (Gongbei Potianyang)	PTY16	109.174782 18.386907
27	PTY (Gongbei Potianyang)	PTY17	109.177542 18.386192
28	PTY (Gongbei Potianyang)	PTY18	109.177678 18.386702
29	PTY (Gongbei Potianyang)	PTY19	109.179279 18.389151
30	PTY (Gongbei Potianyang)	PTY20	109.171181 18.381643
31	PTY (Gongbei Potianyang)	PTY21	109.170959 18.381306
32	PTY (Gongbei Potianyang)	PTY22	109.170933 18.380960
33	PTY (Gongbei Potianyang)	PTY23	109.172236 18.380812
34	PTY (Potianyang)	PTY25	109.171872 18.384848
35	PTY (Potianyang)	PTY26	109.171735 18.385809
36	PTY (Potianyang)	PTY27	109.171334 18.385613
37	PTY (Potianyang)	PTY28	109.176675 18.390892
38	PTY (Potianyang)	PTY29	109.576224 18.391316
39	PTY (Potianyang)	PTY30	109.176572 18.391628
40	PTY (Chicao Potianyang)	PTY31	109.165474 18.394972
41	PTY (Chicao Potianyang)	PTY32	109.169416 18.400351
42	PTY (Chicao Potianyang)	PTY33	109.177125 18.403360

43	PTY (Dabang Potianyang)	PTY34	109.178099 18.403490
44	PTY (Dabang Potianyang)	PTY35	109.178365 18.402851
45	PTY (Damao Potianyang)	PTY36	109.179946 18.403716
46	PTY (Damao Potianyang)	PTY37	109.180903 18.404956
47	PTY (Damao Potianyang)	PTY38	109.178484 18.403882
48	PTY (Damao Potianyang)	PTY39	109.190620 18.405818
49	PTY (Damao Potianyang)	PTY40	109.188900 18.405587
50	RSGL (Reshuigong Road)	RSGL5	109.188900 18.405587
51	RSGL (Reshuigong Road)	RSGL6	109.158736 18.383122
52	RSGL (Reshuigong Road)	RSGL7	109.158511 18.383627
53	RSGL (Reshuigong Road)	RSGL8	109.158279 18.388935
54	MD (Madan)	MD4	109.157831 18.382921
55	MD (Madan)	MD5	109.160029 18.383564
56	MD (Madan)	MD6	109.157677 18.380761
57	NF (Nanfan)	NF3	109.158009 18.380106
58	NF (Nanfan)	NF4	109.146873 18.383495
59	NF (Nanfan)	NF5	109.145759 18.384789
60	BT (Batou)	BT1	109.142166 18.382498
61	BT (Batou)	BT2	109.142546 18.381577
62	BT (Batou)	BT3	109.139532 18.384585
63	SM (Shamai)	SM1	109.138267 18.384064
64	SM (Shamai)	SM2	109.139817 18.384317
65	SM (Shamai)	SM3	109.153759 18.381424
66	SM (Shamai)	SM4	109.152867 18.380587
67	SM (Shamai)	SM5	109.151202. 18.380501
68	SM (Shamai)	SM6	109.151228 18.380074
69	SM (Shamai)	SM7	109.151171 18.379657
70	SM (Shamai)	SM8	109.150444 18.379546
71	SM (Shamai)	SM9	109.150125 18.379159
72	SM (Shamai)	SM10	109.149695 18.376828
73	NF (Nanfan)	NF6	109.149838 18.377091
74	NF (Nanfan)	NF7	109.150513 18.378629
75	NF (Nanfan)	NF8	109.153409 18.378597
76	NF (Nanfan)	NF9	109.152426 18.378505
77	NF (Nanfan)	NF10	109.151908 18.378973
78	NF (Nanfan)	NF11	109.149271 18.378298
79	NF (Nanfan)	NF12	109.149075 18.377828
80	NF (Nanfan)	NF13	109.149956 18.378421
81	NF (Nanfan)	NF14	109.152975 18.380625
82	NF (Nanfan)	NF15	109.149589 18.378900
83	CX (Chengxi)	CX1	109.146855 18.376863
84	CX (Chengxi)	CX2	109.147263 18.376790
85	CX (Chengxi)	CX3	109.147611 18.376945
86	CX (Chengxi)	CX4	109.147187 18.377289

87	CX (Chengxi)	CX5	109.146275 18.377093
88	CX (Chengxi)	CX6	109.146298 18.376494
89	CX (Chengxi)	CX7	109.145022 18.376238
90	CX (Chengxi)	CX8	109.145840 18.375238
91	CX (Chengxi)	CX9	109.146343 18.379510
92	SM (Shamai)	SM11	109.134327 18.383388
93	SM (Shamai)	SM12	109.135732 18.385932
94	SM (Shamai)	SM13	109.136576 18.383925
95	SM (Shamai)	SM14	109.136255 18.383425
96	SM (Shamai)	SM15	109.136202 18.383691
97	SM (Shamai)	SM16	109.135786 18.384160
98	SM (Shamai)	SM17	109.135687 18.384530
99	SM (Shamai)	SM18	109.135271 18.384588
100	HT (Haitang)	HT1	109.135973 18.384014
101	HT (Haitang)	HT2	109.133750 18.382654
102	HT (Haitang)	HT3	109.133019 18.383226
103	HT (Haitang)	HT4	109.132143 18.383382
104	HT (Haitang)	HT5	109.131502 18.383660
105	HT (Haitang)	HT6	109.132569 18.384065
106	HT (Haitang)	HT7	109.131847 18.384050
107	HT (Haitang)	HT8	109.131268 18.384619
108	HT (Haitang)	HT9	109.131027 18.384027
109	HT (Haitang)	HT10	109.130192 18.384212
110	GB (Gongbei)	GB2	109.162099 18.381965
111	GB (Gongbei)	GB3	109.162019 18.380207
112	GB (Gongbei)	GB4	109.161505 18.386650
113	DM (Damao)	DM1	109.177149 18.403364
114	DM (Damao)	DM2	109.180017 18.403630
115	DM (Damao)	DM3	109.178293 18.404067
116	DM (Damao)	DM4	109.185294 18.403425
117	DM (Damao)	DM5	109.184470 18403215
118	DM (Damao)	DM6	109.185478 18.403384
119	DM (Luoji)	DM7	109.190157 18.419816
120	BL (Beiling)	BL1	109.182269 18.419855
121	BL (Beiling)	BL2	109.183372 18.429977
122	BL (Beiling)	BL3	109.128402 18.384170
123	BL (Beiling)	BL4	109.127282 18.383685
124	BL (Beiling)	BL5	109.126159 18.382335
125	BL (Beiling)	BL6	109.126240 18.381531
126	BL (Beiling)	BL7	109.126351 18.379593
127	BL (Beiling)	BL8	109.125408 18.379476
128	BL (Beiling)	BL9	109.125788 18.378215
129	BL (Beiling)	BL10	109.126459 18.384159
130	BL (Beiling)	BL11	109.153373 18.384905

131	BL (Langdian)	BL12	109.182863 18.416750
132	MD (Madan)	MD7	109.153044 18.384894.
133	MD (Madan)	MD8	109.153359 18.385633
134	MD (Madan)	MD9	109.153244 18.385234
135	MD (Madan)	MD10	109.151840 18.384834
136	MD (Madan)	MD11	109.151165 18.385158
137	MD (Madan)	MD12	109.150780 18.384884
138	MD (Madan)	MD13	109.155668 18.386661
139	MD (Madan)	MD14	109.155685 18.387191
140	MD (Madan)	MD15	109.155707 18:387421
141	MD (Madan)	MD16	109.155417 18.387810
142	MD (Madan)	MD17	109.154519 18.387436
143	MD (Madan)	MD18	109.154843 18.388180
144	MD (Madan)	MD19	109.157161 18.387790
145	LF (Lifan)	LF1	109.215252 18.413615
146	LF (Lifan)	LF2	109.215649 18.432390
147	LF (Lifan)	LF3	109.215190 18.432706
148	BG (Baogu)	BG1	109.217692 18.412543
149	BG (Baogu)	BG2	109.218079 18.413152
150	BG (Baogu)	BG3	109.216335 18.409233
151	BG (Baogu)	BG4	109.209041 18.400740
152	BG (Baogu)	BG5	109.207755 18.399408
153	BG (Baogu)	BG6	109.206666 18.399825
154	BG (Baogu)	BG7	109.206689 18.399438
155	BG (Baogu)	BG8	109.204816 18.397948
156	BG (Baogu)	BG10	109.206228 18.397588
157	NB (Nanbin)	NB1	109.203599 18.395999
158	NB (Nanbin)	NB2	109.203213 18.395683
159	NB (Nanbin)	NB3	109.202543 18.394472
160	NB (Nanbin)	NB4	109.194033 18.380360
161	NB (Nanbin)	NB5	109.193919 18.378574
162	NB (Nanbin)	NB6	109.178587 18.350777
163	NB (Nanbin)	NB7	109.179871 18.352113
164	NB (Nanbin)	NB8	109.191427 18.372887
165	NB (Nanbin)	NB9	109.192370 18.373202
166	NB (Nanbin)	NB10	109.192177 18.372175
167	NB (Nanbin)	NB11	109.193635 18.373637
168	NB (Nanbin)	NB12	109.193159 18.372880
169	NB (Nanbin)	NB13	109.194269 18.374049
170	YA (Yaan)	YA1	109.195585 18.349518
171	YA (Yaan)	YA2	109.195005 18.349235
172	YA (Yaan)	YA3	109.185325 18.351019
173	YA (Yaan)	YA4	109.184711 18.351264
174	YA (Yaan)	YA5	109.184356 18.349183

175	YA (Yaan)	YA6	109.184356 18.349183
176	TC (Taice)	TC1	109.087432 18.373135
177	TC (Taice)	TC2	109.086007 18.373159
178	TC (Taice)	TC3	109.086137 18.373511
179	TC (Taice)	TC4	109.091878 18.372789
180	FL (Fengling)	FL1	109.083069 18.370051
181	FL (Fengling)	FL2	109.078757 18.371723
182	FL (Fengling)	FL3	109.078002 18.370123
183	FL (Fengling)	FL4	109.077213 18.371941
184	FL (Fengling)	FL5	109.077356 18.372439
185	FL (Fengling)	FL6	109.067801 18.371039
186	SG (Sangeng)	SG1	109.052616 18.381189
187	SG (Sangeng)	SG2	109.055611 18.381315
188	FL (Fengling)	FL7	109.078067 18.372389
189	FL (Fengling)	FL8	109.077302 18.372862
190	SG (Meidong)	SG3	109.027401 18.369391
191	BP (Baoping)	BP1	109.132956 18.372694
192	BP (Baoping)	BP2	109.133682 18.372106
193	HT (Haitang)	HT11	109.119130 18.381149
194	HT (Haitang)	HT12	109.126221 18.385847
195	HT (Haitang)	HT13	109.125415 18.385113
196	HT (Haitang)	HT14	109.119711 18.376584
197	HT (Haitang)	HT15	109.124029 18.319708
198	HT (Haitang)	HT16	109.123165 18.381320
199	SN (Shuinan)	SN1	109.160618 18.357968
200	SN (Shuinan)	SN2	109.158213 18.359397
201	SN (Shuinan)	SN3	109.158434 18.360591
202	SN (Shuinan)	SN4	109.156689 18.359926
203	SN (Shuinan)	SN5	109.157543 18.360745
204	SN (Shuinan)	SN6	109.158185 18.361324
205	SN (Shuinan)	SN7	109.155561 18.358472
206	SN (Shuinan)	SN8	109.154712 18.357320
207	SN (Shuinan)	SN9	109.154996 18.359741
208	SN (Shuinan)	SN10	109.153026 18.362403
209	SN (Shuinan)	SN11	109.154081 18.361506
210	BP (Gangmen)	BP4	109.125084 18.364401
211	BP (Gangmen)	BP5	109.125355 18.366059
212	BP (Baoping)	BP3	109.100770 18.375725
